# Ultrasound of the infant hip: manual fixation is equivalent to Graf’s technique regarding image quality—a randomized trial

**DOI:** 10.1186/s12887-019-1392-z

**Published:** 2019-01-10

**Authors:** Peter Voitl, Christian Sebelefsky, Sara Hosner, Astrid Woditschka, Susanne Diesner, Andreas Böck

**Affiliations:** 1 First Vienna Pediatric Medical Center, Donau-City-Straße 1, 1220 Vienna, Austria; 2 Sigmund Freud Private University, Vienna, Austria; 3 Department of Pediatrics and Adolescent Medicine, Medical University of Vienna, Vienna, Austria

**Keywords:** Ultrasonography, Musculoskeletal system, Infant, Hip dislocation, Mass screening

## Abstract

**Background:**

In Middle Europe ultrasonography is the standard method used to screen for developmental dysplasia of the hip in infants. Our aim was to determine whether manual fixation of the child is equivalent to Graf’s technique regarding image quality.

**Methods:**

This randomized trial was conducted at a free-standing general pediatric outpatient clinic in Vienna, Austria. Healthy infants in the 1st and between the 6th and 8th week of life with no hip malalignment were included. After randomization, Group 1 was examined using Graf’s fixation device and participants in Group 2 were fixated on the examination couch by their parents. In a second step, all images underwent a blinded evaluation.

**Results:**

A total of 117 babies (Group 1: *n* = 62, Group 2: *n* = 54, excluded: *n* = 1) were examined and 230 images (Group 1: *n* = 122, Group 2: *n* = 108) were evaluated, of which 225 were sonographically normal. Two images, showing a type IIa right hip and a type IIa + left hip respectively, were excluded. One participant had to be excluded as the respective images showed two pathologic hip joints. Two images in Group 1 and three in Group 2 were not evaluable. No statistical association between image quality (11 quality criteria and overall evaluability) and fixation technique (0.12 ≤ *p* ≤ 1.0 or constant) was found.

**Conclusions:**

Considering sonographically normal hip joints, we found no evidence that manual fixation differed from Graf’s technique regarding image quality. In future studies, hip pathologies should be included and discomfort of infants and parents during the examination should be addressed.

**Trial registration:**

German Clinical Trials Register, ID: DRKS00015694), registered retrospectively on October 7th, 2018.

## Background

### Ultrasound of the infant hip

Over the last decades ultrasound has become the standard procedure used for screening of the infant hip. This is owed to the low specificity of clinical examination in detecting developmental dysplasia of the hip (DDH) [1]. For a variety of reasons sonography is also superior to former diagnostic methods such as radiography or arthrography [2–4]. In addition, hip ultrasound screening has proven to reduce surgical procedures in infants with DDH, hospitalization rates and the likelihood of late presentations [5–7]. Rosendahl et al. [8] showed that proactive sonographic surveillance is also capable of reducing overtreatment. Thereby, treatment costs and parental anxiety can be reduced.

Since the first introduction of the ultrasound screening in infants for early detection of DDH several studies have been initiated to gather currently applied methods. In 2014, the heads of 31 orthopedic departments in German hospitals participated in a study surveying the examination techniques and instruments applied for hip ultrasound imaging [9]. A fixation device was used in 100% of the hospitals and 35.5% used a fixated ultrasound probe as recommended by Graf. In 2011 a study group from the Netherlands surveyed members of the Dutch Paediatric Orthopaedic Society (DPOS) in order to gather diagnostic and therapeutic procedures used in DDH [10]. The Graf classification was used in 78%, while the remaining 22% used the femoral head for making a diagnosis. To the extent of our knowledge, fixation devices are commonly used in Europe, but their use is less widespread in North America.

In 1992, ultrasonography of the infant hip was established as the standard screening method in Austria and subsequently incorporated into the Mutter-Kind-Pass (MKP), the official Austrian document for medical follow-up of pregnancy and child development [2, 11]. In 1996, ultrasonography was also approved as the standard screening procedure in Germany and in the following year in Switzerland [2].

### Graf’s hip ultrasound technique

In 1980 Graf et al. [12] described an approach of diagnosing congenital hip-joint dislocations by means of ultrasonography. Throughout the years they developed a standardized technique on how to position an infant in order to obtain images which are reproducible, reliable and independent of examiner skill and experience [2]. According to these guidelines, the child is positioned sideways in an elastic fixation device which prevents them from kicking and keeps them in a fixated position. The ultrasound probe is likewise fixated in a vertical position, merely allowing limited movement. According to Graf et al. [13, 14] hip joints are classified into four main types.

### Objectives

The primary objective of this study was to determine whether manual fixation of the infant is equivalent to Graf’s fixation technique regarding the quality of hip ultrasound images. To our knowledge, this is the first attempt to address this question. In addition, further variables with a potential influence on the evaluability of hip ultrasound images were examined.

## Methods

### Study design and participants

This randomized trial was initiated to compare two techniques of hip ultrasound imaging using blinded evaluation of images. One group of infants was examined using Graf’s fixation device (Group 1) and the other group was being fixated on the examination couch by their parents or legal guardians during the examination (Group 2). The study was conducted from September 2014 to November 2015 at the First Vienna Pediatric Medical Center (FVPMC), a free-standing general pediatric outpatient clinic in Vienna, Austria.

Healthy female and male newborns and babies up to 8 weeks were included in the study. Participants diagnosed with hip dysplasia or any other malalignment, regardless of the respective classification level, were excluded and the corresponding images were discarded.

Parents were recruited during usual MKP visits by the examining doctor (AW), who also randomized the study participants. A number was blindly drawn from a box with 300 small paper cards of which 150 were labeled with the number “1” and 150 with the number “2”. According to this number children were assigned either to Group 1 or 2.

Initially, we planned to include 150 participants and 300 images in the study. After having examined more than two thirds of the initially scheduled participants (*n* = 117), we decided to evaluate all images obtained by then in order to gain a first impression of their quality. Following a preliminary statistical analysis of the data, we came to the conclusion that further images would not enhance the statistical power of our results and therefore finished data collection. This decision was made after careful consideration and being mindful of the unequal distribution of infants to Group 1 and 2. In order to achieve a power of 0.8 at an alpha of 0.05, one would need a total sample size of 5322 images. If the power calculation had shown that a difference between the groups could be proven well with *n* = 300, then this could have been interpreted as a weak point of the study (since the actual number of images was somewhat smaller (*n* = 230)). Here, the required number of images is much larger. This can justify the fact that a significantly smaller number of images were used.

### Ultrasound examination: Positioning techniques and equipment

Corresponding to the guidelines in the MKP, the ultrasound examinations were conducted within the first week of life and/or between the sixth and eighth week. All examinations were carried out by one member of the research team (AW). The collected images were saved for subsequent evaluation and all necessary data were entered into an Excel table. This file contained all primary parameters relating to the evaluability of images. The following secondary parameters were also collected:

- Participant number
- Group
- Parents’ assistance
- Sex
- Age in days (birthdate)
- Duration of the examination (< 5 min; 5–10 min, 10–15 min, 15–20 min)
- Premature birth

#### Positioning techniques

**Fig. 1:**
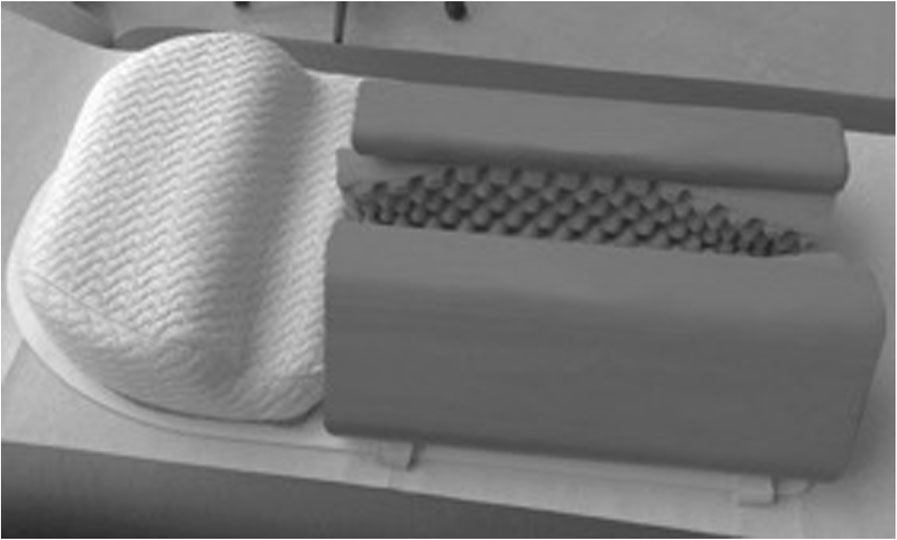
Graf’s fixation device as used in Group 1

**Fig. 2:**
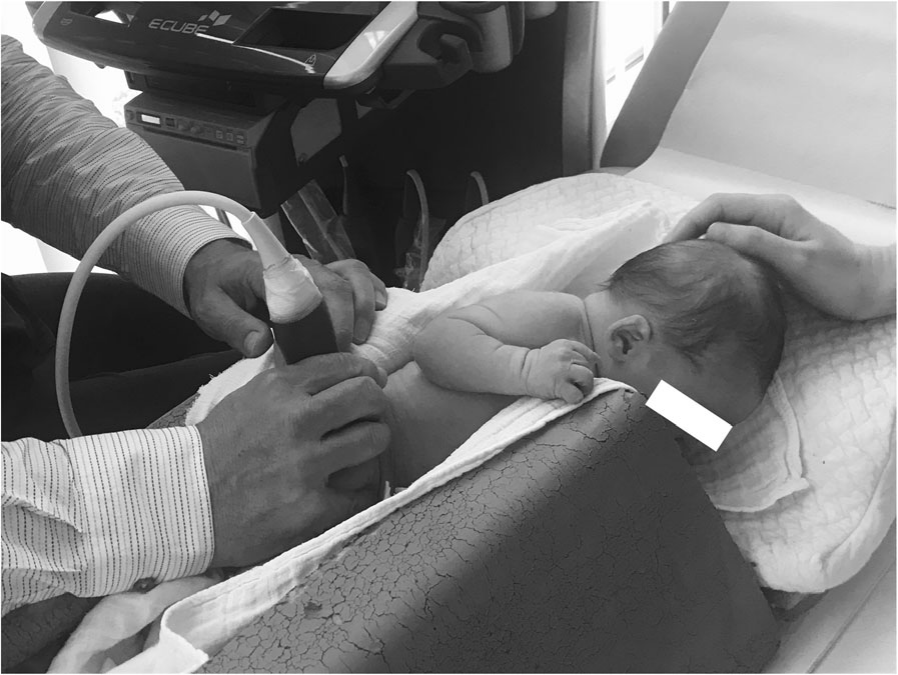
Graf’s fixation device as used in Group 1 (with baby in it)

Babies assigned to Group 1 were examined according to Graf’s principles using a foam shell [2]. This shell consists of a pillow-like head section and a lower fixating part with a lengthwise gap (Fig. 1) which can be spread apart to position the baby (Fig. 2). A cloth diaper is placed on the fixation device, allowing it to hang into the gap like a hammock. Depending on the baby’s weight, the diaper can be loosened or tightened to the needs of the examining doctor, just so the baby’s hip slightly protrudes the edges of the fixation device. The baby’s legs should not be stretched to promote better fixation and stay in their natural slightly flexed position to avoid excessive rotation of the hip. The knee joint should not protrude from the fixation device, so as not to turn the greater trochanter dorsally and, hence, hinder the scanning process.

According to Graf’s original principles [2], the examiner is supposed to conduct the ultrasound scan in a standing position and hold the fixated probe in both hands. In our setting, the examinations were conducted in a sitting position with the probe only being manually fixated by the examiner. This was due to empirical experience and the expectation that no difference in image quality would result from this approach.

**Fig. 3:**
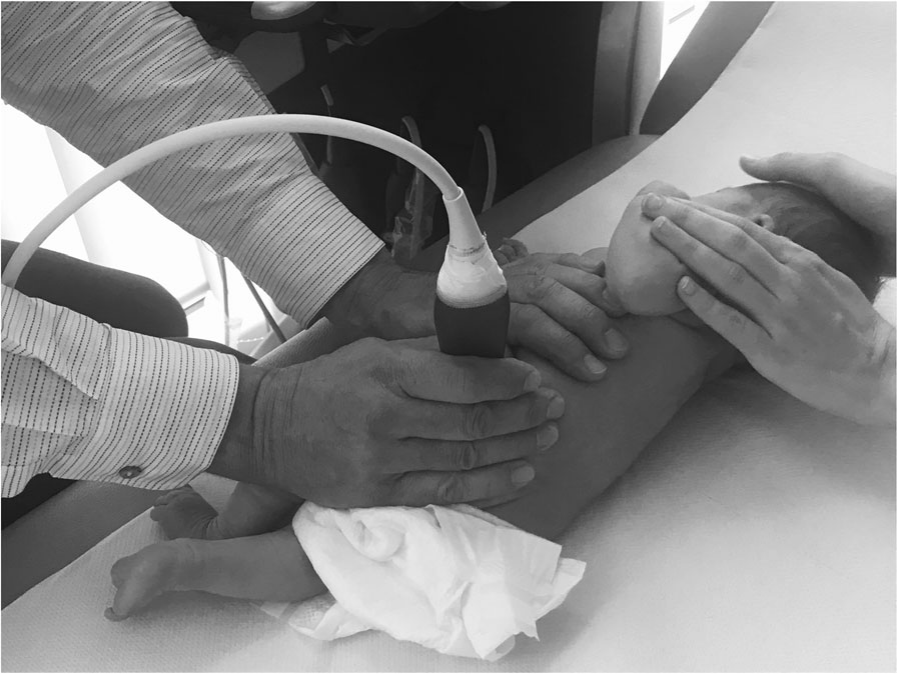
Manual fixation of an infant as applied in Group 2

In Group 2, the newborns were placed on the examination couch and gently rolled over to a sideways position. The parent was advised to hold the baby and its legs to prevent it from rolling over (Fig. 3).

In the Excel table the examining doctor indicated whether help was needed from parents during the scanning process due to agitation or restlessness of the baby. This assistance mainly included manual fixation of the legs and the upper body to prevent rotation.

#### Ultrasound scanners

Two ultrasound scanners were used in the course of this study: the Ambisea ComboScan HD, manufactured in 2010, an all-in-one colour Doppler scanner with integrated PC and LCD and Ultra-frequencies between 2 and 10 MHz [15] and the Alpinion E-CUBE 9 DIAMOND, manufactured in 2012, with a single-crystal transducer technology and Ultra-frequencies between 1 and 17 MHz [16].

### Evaluation of ultrasound images

The ultrasound images obtained in both groups were evaluated by another member of the research team (PV) as soon as all examinations had been conducted. This assessment was done without knowledge of the applied method and was therefore blinded. For each quality criterion as well as the overall evaluability “evaluable” or “not evaluable” was entered into the mentioned Excel file. The overall evaluability denotes whether an image is generally usable for determining the proper alignment of hip joints and whether it is suitable for diagnosing acetabular dysplasia.

#### Quality criteria: Anatomical structures and landmarks

The Excel table contained the criteria for evaluation of the ultrasound images (Table 1). For each of the 11 quality criteria and for the overall evaluability “evaluable” or “not evaluable” were indicated. In addition, it was indicated whether anomalies exist and, if so, these were specified.

**Table 1:** Evaluated quality criteria and the overall evaluability of all images

Quality criteria *All images*	Fixation device *Evaluable*	No fixation device *Evaluable*	Fixation device *Not evaluable*	No fixation device *Not evaluable*	Total no. of *evaluated images*
Landmark 1	118 (96.7%)	107 (99.1%)	4 (3.3%)	1 (0.9%)	230
Landmark 2	108 (88.5%)	96 (88.9%)	14 (11.5%)	12 (11.1%)	230
Landmark 3	118 (96.7%)	107 (99.1%)	4 (3.3%)	1 (0.9%)	230
Chondroosseous border	122	108	0	0	230
Femoral head	122	108	0	0	230
Synovial fold	122	108	0	0	230
Joint capsule	122	108	0	0	230
Concavity-convexity	122	108	0	0	230
Bony roof line	118 (96.7%)	107 (99.1%)	4 (3.3%)	1 (0.9%)	230
Base line	109 (89.3%)	96 (88.9%)	13 (10.7%)	12 (11.1%)	230
Cartilaginous roof line	122	108	0	0	230
Overall evaluability	120 (98.4%)	105 (97.2%)	2 (1.6%)	3 (2.8%)	230

**Fig. 4:**
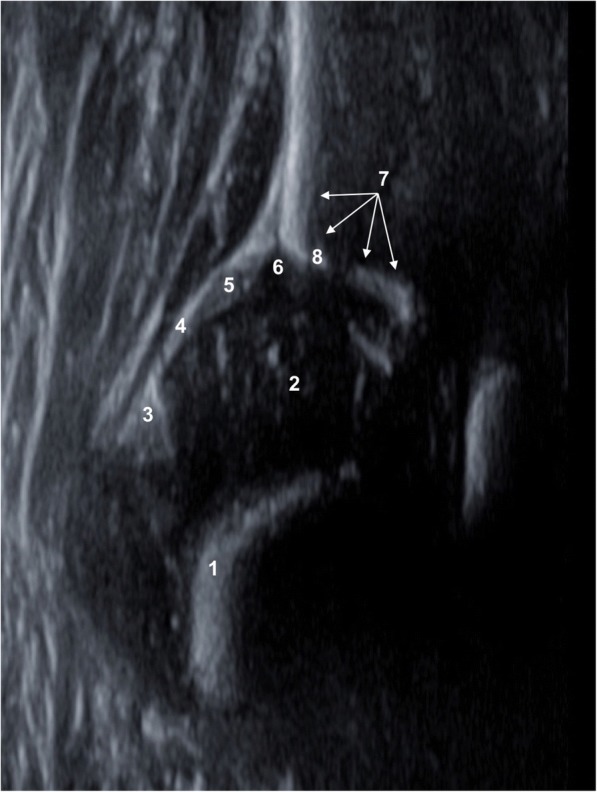
Anatomical structures: chondroosseous border (1), femoral head (2), synovial fold (3), joint capsule (4), labrum acetabulare (5), cartilagineous roof (6), bony roof (7), bony rim (concavity-convexity) (8)

**Fig. 5:**
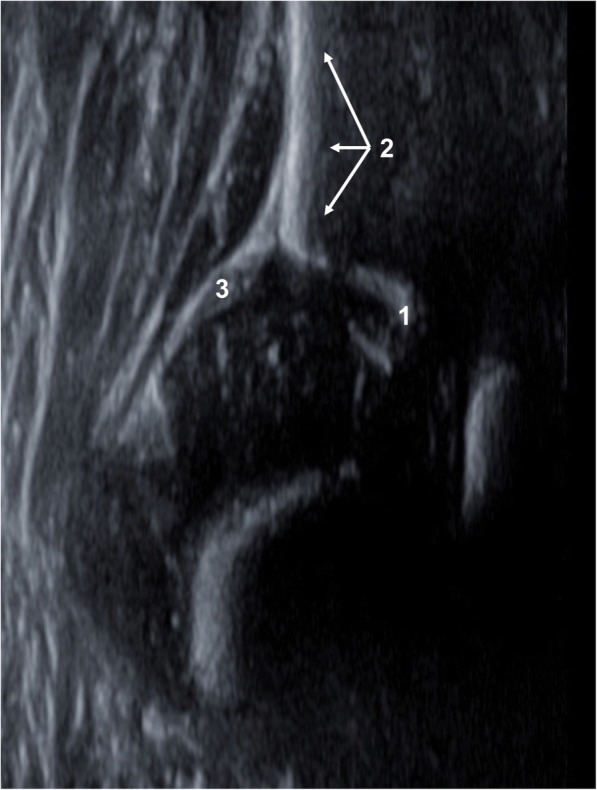
Landmarks: lower limb of os ilium (1), midsection of bony acetabular roof (2), cartilaginous acetabular labrum (3)

The first step to make an accurate diagnosis of the infant hip is to identify the anatomical structures (Fig. 4). Following this, three landmarks need to be visualized in the same ultrasound image ensuring it to be in the true coronal plane of the hip joint [2, 17] (Fig. 5). This allows a correct assessment of the hip joint. The landmarks are to be identified according to their numbering.

#### α and β angle and the classification of hip joints

**Fig. 6:**
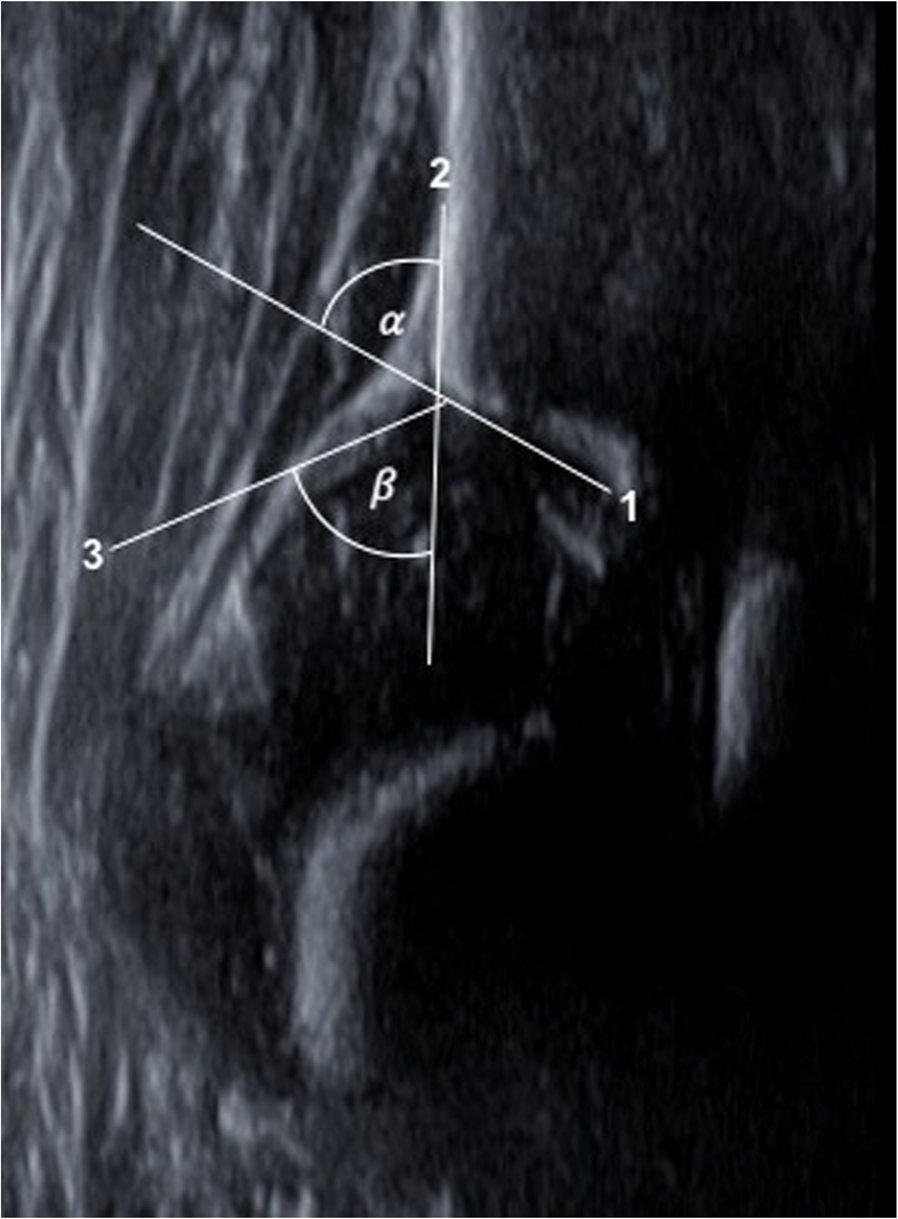
Bony roof line (1), base line (2), cartilage roof line (3), alpha and beta angle

The α angle (bony roof angle) and the β angle (cartilage roof angle) are essential parameters to classify infant hip joints according to Graf [2, 17]. These are to be determined in the standard plane. This measurement is independent of the baby’s position, the position of the femoral head or the projection. In order to define both angles, three measurement lines need to be drawn on the ultrasound image, which rarely intersect in one point (Fig. 6).

According to Graf et al. [13, 14] the alignment of hip joints is characterized using a classification based on the measurement of α and β angles, with type I and II indicating centered, type III and IV decentered hip joints and type D a joint which is about to decenter.

This classification was also applied in our study. Only images of hip joints classified as type I were used for assessment of the ultrasound techniques. Images of hip joints classified as type II or higher were excluded.

### Hip ultrasonography experience of investigators

All ultrasound examinations were conducted by AW, a pediatric resident with approximately 500 ultrasound examinations of experience, who completed her hip ultrasound training in 2014. The images obtained were evaluated by a certified pediatric consultant (PV), who completed his infant hip ultrasound training in 1995 and his medical training in pediatrics in 1996. He is the head of the FVPMC and a ÖGUM (Austrian Society for Ultrasound in Medicine) certified trainer in pediatric ultrasound with approximately 17,700 ultrasound examinations of experience.

### Statistical methods

After collection of the data with Excel 2016, the statistical analysis was performed using IBM SPSS Statistics Version 21. For each of the primary and secondary parameters frequencies were calculated. For statistical testing, Fisher’s exact test (two-sided exact significance) was used and the significance level was set to 5%. The power calculation was performed using G*Power 3.1.9.2 [18].

## Results

### Evaluation of ultrasound images

Descriptive results pertaining to the quality criteria and the respective assessments of all 230 evaluated hip ultrasound images can be found in Table 1. There were no significant differences in overall evaluability, when comparing left to right hip images (*p* = 0.37).

Table 2 contains the *p*-values of the Fisher’s exact test for the evaluability of quality criteria and the group assignment, both for right and left hip images. Only three of the evaluated eleven quality criteria and the overall evaluability are shown, as the remaining parameters were evaluable in all of the cases and therefore constants or showed p-values of 1.0.

**Table 2:** Results pertaining to cross tabulations of quality criteria with evaluability of right and left hip images and group assignment (Exact Fisher’s Test, p-values)

Quality criteria	Cross tabulation quality criteria (evaluable/not evaluable) / Group (1/2) (*p*-values, Fisher’s exact test)	
	Left hip	Right hip
Landmark 1	0.12	0.47
Landmark 3	1.0	0.50
Bony roof line	0.12	0.47
Overall evaluability	0.47	1.0

### Study participants

A total of 117 babies were examined in the course of this study. There was no withdrawal from participation. Two study participants had one pathologic hip each and, therefore, the respective images were excluded from statistical analysis, whereas the images of the other hip were included. One participant had a type IIa right hip, and another infant had a type IIa + left hip. One participant had to be excluded during the evaluation phase as the respective images showed two pathologic hip joints. Of the 116 infants eligible for image assessment 62 belonged to Group 1 and 54 to Group 2. Hence, a total of 230 hip ultrasound images, with 122 belonging to Group 1 and 108 to Group 2, were included in this study.

There were no differences between infants examined in the 1st compared to the 6th to 8th week of life regarding sex (*p* = 0.098), prematurity (*p* = 1.0), fixation type (p = 1.0), needed assistance (*p* = 1.0), duration of the examination (*p* = 1.0), and the overall evaluability of images (*p* = 1.0 for both left and right hips).

#### Gender distribution

Of the 116 participants 50 (43.1%) were male and 66 (56.9%) were female babies. Group 1 consisted of 37 (59.7%) girls and 25 (40.3%) boys and Group 2 of 29 (53.7%) girls and 25 (46.3%) boys.

#### Age distribution

**Fig. 7:**
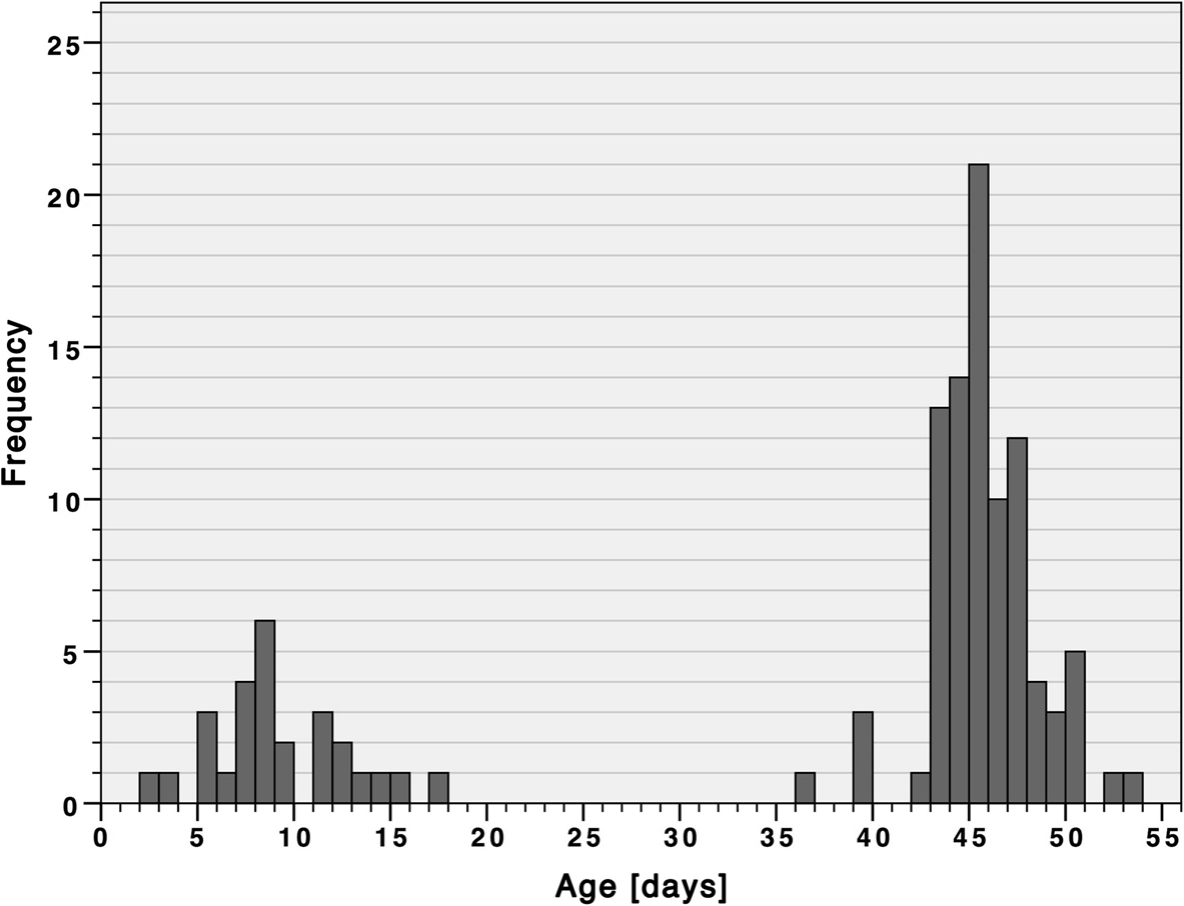
Age distribution of study participants (*n* = 116)

The age distribution of included infants (*n* = 116) showed a minimum of 2 and a maximum of 53 days with a mean of 36.8 and a standard deviation of 15.79 days (Fig. 7). The mean age of children in Group 1 was 35.34 days (standard deviation: 16.46) and in Group 2 38.48 days (standard deviation: 14.96). Figure 7 shows two peaks in the age distribution. This is due to the recommendations in the MKP, involving one examination during the first and between the sixth and eighth week of life. Usually the first hip ultrasound examination is conducted at the hospital where the baby was born. Therefore, most children examined in the FVPMC were between 36 and 56 days of age.

#### Prematurity

Nine children were premature babies, born between the 33rd and 37th gestation week. Four of them were twins, born in gestation week 33 + 4 and 36 + 6. The remaining five premature babies were singletons. We did not find a statistically significant influence of prematurity on the outcome of image quality assessments (quality criteria: 0.39 ≤ *p* ≤ 1.0, overall evaluability: left hip images: *p* = 1.0, right hip images: *p* = 0.28).

### Assistance of parents and duration of examinations

In Group 1 90.3% (56/62) of the infants needed no manual fixation in addition to the foam shell during the examination, while 9.7% (6/62) had to be fixated additionally. Of the 54 babies in Group 2, three (5.6%) needed no fixation at all, whereas 51 (94.4%) had to be additionally held by a parent. Infants not fixated in a foam shell were far more likely to need manual fixation of their parents (*p* < 0.001). There is no statistically significant association between the provided assistance and the outcome of image quality assessments (quality criteria: 0.12 ≤ *p* ≤ 1.0, overall evaluability: left hip images: *p* = 0.50, right hip images: *p* = 0.62).

Of the 116 study participants 114 were scanned on both hips in less than 5 min. The remaining two infants, both belonging to Group 1, required between 5 and 10 min of examination time. There was no statistically significant association between the duration of examinations and the participation in Group 1 or 2 (*p* = 0.50). We could not determine a statistically significant association between examination durations and image quality assessments (quality criteria: either constants or *p* = 1.0, overall evaluability: left hip images: *p* = 1.0, right hip images: *p* = 1.0).

## Discussion

We aimed to provide evidence for the empirical observation that fixation of infants according to Graf et al. [2] is not required for quality imaging. According to our expectations, we found no statistical association between image quality (indicated by quality criteria) and the used positioning technique. These findings are furthermore supported by the results regarding the overall evaluability of images. Two ultrasound images in Group 1 (with fixation device) and three in Group 2 (without fixation device) could not be assessed appropriately. For all five images, this was due to accidental tilting of the probe. Technical improvements in ultrasound probes may explain why a fixation apparatus is not as necessary as in the past.

In Group 2 (without fixation device) 51 study participants (94.4%) had to be fixated manually by parents during the ultrasound examination. In Group 1 (with fixation device) this was only the case in 9.7% (6/62). If neither a fixation apparatus nor manual fixation is used, obtaining a quality ultrasound image is impossible in the majority of cases. It has to be taken into consideration if babies in Group 2 needed forceful fixation or were just gently held, which we did not gather.

Graf’s fixation device is widely used in German-speaking countries and other states in Middle Europe, whereas fixating the ultrasound probe is not as widespread. According to Peterlein et al. [9] a cradle for positioning the infant is used in 100% of the surveyed hospitals, whereas only 35.5% use a fixated ultrasound probe. A standardized examination method, as described by Graf et al. [2], is a reliable approach to guarantee the reproducibility of quality ultrasound images independent of the examiner’s skill. Nevertheless, in our study we were able to obtain quality images only by instructing parents to fixate infants on the examination couch and not using a fixated ultrasound probe.

From the examiner’s perspective, both positioning techniques applied in this study were tolerated well on the part of infants and parents. The examining pediatrician reported having had no issues in applying both methods. Even though Graf’s fixation device should make further positioning assistance unnecessary, additional manual fixation is needed in some cases. We are aware that at several other institutions the sonographer is able to perform the ultrasound alone. This is done by stabilizing the baby with one hand and using the other to hold the probe. In addition, a foot pedal is used to save images, which does not require parental involvement. Therefore, one advantage of the positioning frame could be to eliminate the need for the “third hand” and reduce dependence on operator skill. Although omission of the foam shell requires more attention and additional manual fixation, we suppose it is more comfortable for infants to be held solely by their parents.

### Limitations

We only included type I hip joints in this study. Designing a study with the intention to evaluate images of hip pathologies would have required a different study design and mode of recruitment of participants, which would have gone beyond the scope of this project. This is mainly due to the low proportion of images showing hip pathologies (2.2% (5/230) in our study). Hence, we can only assume but not prove that hip pathologies are detected with similar precision if no fixation device is used. Further research is needed to provide evidence for this assumption. We did not gather data on discomfort of infants (e.g., cry counts or duration) or parents (e.g., post-scan survey) during the ultrasound examination. We neither recorded the exact level of assistance needed to fixate the baby. Comparing groups with and without rigorous fixation with respect to these parameters is worth investigating in future studies. This also applies to older and more active babies. Time slots for ultrasound examinations (< 5 min, 5–10 min) were only gathered using a nominal scale. Measuring the elapsed time more precisely and therefore metrically might be beneficial with respect to future studies and future study objectives. Although we were able to produce quality ultrasound images without rigorous fixation of infants, our findings resulted from a single center investigation. We do not assume but cannot rule out that different examiners and equipment might lead to differing results. There was no direct comparison of images and parental or sonographer preference in the same patient. A study design in which both types of scanning are performed in each patient, with direct comparison of alpha angles and images obtained, might be instructive as to relative ranges of measurement variability; although more complex to perform. Finally, the use of two different ultrasound scanners might also have introduced potential confounding.

## Conclusions

Regarding intact infant hip joints, we could prove that ultrasound imaging solely with manual fixation is an effective approach to obtain quality images. We conclude from our findings that it is equivalent to Graf’s technique. In addition to it, we deem this method to be more gentle. In a general pediatric outpatient clinic like ours with busy schedules and the time factor playing an omnipresent role, we therefore favor this positioning technique.

Future studies should address the degree of needed assistance for fixation of infants as well as the level of discomfort of infants and the accompanying person during the ultrasound examination. A multicenter approach and the inclusion of hip pathologies would furthermore be beneficiary. We have no evidence that the need for parents’ assistance during the examination or prematurity of infants is linked to the evaluability of ultrasound images.

## Abbreviations

DDH: Developmental dysplasia of the hip
FVPMC: First Vienna Pediatric Medical Center
MKP: Mutter-Kind-Pass (official Austrian document for medical follow-up of pregnancy and child development)
